# Highlight report: Intratumoral metabolomic heterogeneity of breast cancer

**DOI:** 10.17179/excli2017-1045

**Published:** 2017-12-22

**Authors:** Regina Stoeber

**Affiliations:** 1 IfADo - Leibniz Research Centre for Working Environment and Human Factors at TU Dortmund, Ardeystr. 67, D-44139 Dortmund, Germany

## ⁯⁯⁯⁯

Recently, Mikheil Gogiashvili and colleagues from TU-Dortmund have published a study about the metabolomics heterogeneity of breast cancer (Gogiashvili et al., 2017[8]). The background of this study is the practically relevant question, whether measurement of a single biopsy is sufficient when analyzing tumors from a cohort of patients. In recent years metabolic profiling by high-resolution magic angle spinning nuclear magnetic resonance spectroscopy has been increasingly used to characterize the metabolome of breast cancer (Sitter et al., 2010[19]; Giskeodegard et al., 2012[7]; Cao et al., 2012[3]; Choi et al., 2012[4]; 2013[5]). However, so far only a single study has addressed the possible influence of metabolic heterogeneity within a single breast tumor (Park et al., 2016[17]). Therefore, the authors performed multi-core sampling of six small specimens from individual tumors and quantified 32 metabolites. Not unexpectedly, the intertumoral differences were larger compared to intratumoral differences (Gogiashvili et al., 2017[8]). More importantly, a random forest- classifier trained on a sample set of individual tumors correctly predicted tumor identity of an additional set of independent cores from the same tumors (Gogiashvili et al., 2017[8]). Therefore, the study shows that despite the intratumoral heterogeneity the analysis of only one or few replicates per tumor can be justified. This is of high relevance, when large cohorts of patients have to be analyzed. 

Currently, the majority of prognostic studies with cancer patients has been performed based on mRNA (Grinberg et al., 2017[10]; 2015[9]; Marchan et al., 2017[15]; Cadenas et al., 2014[2]; Ghallab et al., 2015[6]; Lohr et al., 2015[14]; Hellwig et al., 2016[13]; Stock et al., 2015[20]; Hammad et al., 2016[11]) or immunostaining (Heimes et al., 2017[12]; Mattsson et al., 2015[16]; Schmidt et al., 2012[18]; Barone et al., 2016[1]). Studies with metabolic profiling by HR MAS ^1^H NMR are still relatively rare in breast cancer. Therefore, the present study of Gogiashvili and colleagues represents an important milestone in this field of research. 
